# Patient outcomes and experience of a task-shared screening and brief intervention service for problem substance use in South African emergency centres: a mixed methods study

**DOI:** 10.1186/s13722-021-00239-5

**Published:** 2021-05-12

**Authors:** Claire van der Westhuizen, Megan Malan, Tracey Naledi, Marinda Roelofse, Bronwyn Myers, Dan J. Stein, Sa’ad Lahri, Katherine Sorsdahl

**Affiliations:** 1 Alan J Flisher Centre for Public Mental Health, Department of Psychiatry and Mental Health, University of Cape Town, 46 Sawkins Road, Rondebosch, Cape Town, 7700 South Africa; 2 Public Health Medicine Division, University of Cape Town, Cape Town, South Africa; 3 Western Cape Department of Health, Cape Town, South Africa; 4 Alcohol Tobacco and Other Drug Research Unit, South African Medical Research Council, Cape Town, South Africa; 5 Department of Psychiatry and Mental Health, University of Cape Town, Cape Town, South Africa; 6 Department of Psychiatry and Neuroscience Institute, SA MRC Unit on Risk and Resilience in Mental Disorders, University of Cape Town, Cape Town, South Africa; 7 Department of Emergency Medicine, University of Cape Town, Cape Town, South Africa; 8 Khayelitsha Hospital Emergency Services, Cape Town, South Africa

**Keywords:** Screening brief intervention and referral to treatment (SBIRT), Task-sharing, Patient experiences, Low- and middle-income countries

## Abstract

**Background:**

Screening, brief intervention and referral to treatment (SBIRT) programmes have resulted in generally positive outcomes in healthcare settings, particularly for problem alcohol use, yet implementation is hampered by barriers such as concerns regarding the burden on healthcare professionals. In low-resourced settings, task-sharing approaches can reduce this burden by using non-professional healthcare workers, yet data are scarce regarding the outcomes and acceptability to patients within a SBIRT service. This study aims to evaluate patient-reported outcomes, patient acceptability, perceived benefits and recommendations for improving a task-shared SBIRT service in South African emergency centres (ECs).

**Methods:**

This mixed methods study incorporates quantitative substance use screening and patient satisfaction data collected routinely within the service at three hospitals, and qualitative semi-structured interviews with 18 EC patient beneficiaries of the programme exploring acceptability and perceived benefits of the programme, as well as recommendations to improve the service. Approximately three months after the acute EC visit, a sub-sample of patients were followed up telephonically to assess patient-reported satisfaction and substance use outcomes.

**Results:**

Of the 4847 patients eligible for the brief intervention, 3707 patients (76%) used alcohol as their primary substance and 794 (16%) used cannabis. At follow-up (n = 273), significant reductions in substance use frequency and severity were noted and over 95% of patients were satisfied with the service. In the semi-structured interviews, participants identified the non-judgemental caring approach of the counsellors, and the screening and psychoeducation components of the intervention as being the most valuable, motivating them to decrease substance use and make other positive lifestyle changes. Study participants made recommendations to include group sessions, market the programme in communities and extend the programme’s reach to include a broader age group and a variety of settings.

**Conclusions:**

This task-shared SBIRT service was found to be acceptable to patients, who reported several benefits of a single SBIRT contact session delivered during an acute EC visit. These findings add to the SBIRT literature by highlighting the role of non-professional healthcare workers in delivering a low-intensity SBIRT service feasible to implement in low-resourced settings.

## Introduction

Screening, brief intervention and referral to treatment (SBIRT) programmes for substance use have been tested in a number of different countries and settings with generally positive results, particularly for interventions targeting problem alcohol use [1–3], yet there is less evidence of benefit for individuals using other drugs [4]. While the evidence for SBIRT has mainly emanated from high-income countries, studies from low-and middle-income countries (LMICs), such as South Africa, have also found promising results [5–7]. Despite the growing body of evidence in support of its benefits, SBIRT is not yet routine practice in many healthcare settings. Barriers to implementation include limited resources, lack of buy-in from leadership, the stigmatisation of people who use substances in healthcare settings, stakeholders’ perceptions of SBIRT acceptability to patients and limited time of health care workers to take on extra tasks [8–10].

A major barrier to the delivery of SBIRT is that many healthcare providers are reluctant to address substance use in a clinical consultation citing fear of patients’ negative reactions to screening and intervention, as well as doubts regarding patient disclosure of risky substance use [11, 12]. While data from LMICs is limited regarding stakeholder views of SBIRT, a recent SBIRT evaluation study was conducted in the Western Cape province of South Africa and stakeholders raised similar concerns regarding acceptability to patients [13]. However, the available studies investigating acceptability of SBIRT among patients in primary care, inpatient and emergency or trauma settings have found mainly positive findings, with patients reporting that they would feel comfortable disclosing substance use, receiving an intervention and being referred for additional services if necessary [14, 15].

A widely reported barrier to SBIRT implementation is the availability of staff who often have many competing priorities, with the provision of usual healthcare services taking precedence over perceived ‘add-ons’, such as SBIRT [9, 16, 17]. Task-sharing has been put forward as a strategy to decrease such demands on healthcare professionals by using other cadres of non-specialist workers to provide care [18]. This strategy has been used successfully to provide mental health and substance use interventions in a variety of settings [5, 19]. A number of these programmes use non-professional healthcare workers with intensive SBIRT training, thus requiring fewer resources than other task-sharing SBIRT models from high-income countries which employ social workers, doctors or nurses as delivery agents [20]. The terms community health workers and facility-based counsellors are often used to describe a certain cadre of non-professional healthcare worker who does not have any health qualification but is trained to deliver a specific low-intensity service. This cost-saving approach is particularly attractive in LMIC settings, such as South Africa, for tackling widespread public health issues [21]. While SBIRT programmes using a task-sharing approach employing nurses or social workers have been found to be effective and acceptable to patients in healthcare settings, few studies have explored patient experiences and the acceptability of community health worker or facility-based counsellor-delivered SBIRT [22–25]. Although patient involvement is increasingly recognised as an important, effective strategy in improving the quality of healthcare services [26], SBIRT implementation studies have focused mainly on uptake amongst healthcare providers and managers, with patient outcome data often limited to substance use screening tool scores [27, 28].

This study aims to address this gap by providing evidence on: (i) patient-reported outcomes of a SBIRT service delivered in emergency centres (EC) within an LMIC; (ii) the acceptability of the SBIRT programme for service users; (iii) beneficial aspects of the programme from the patients’ view and (iv) recommendations for improving SBIRT delivered in EC settings.

## Methods

### SBIRT programme

In 2016, in response to the burden of substance-related harms [29], the Western Cape provincial government in South Africa adopted a SBIRT programme for delivery in hospital and community health centre ECs, labelling the initiative the Teachable Moment programme. The evidence-based blended motivational interviewing/problem-solving therapy intervention was implemented in three demonstration sites chosen as they serve areas with high levels of substance-related injuries and violence. The intervention consists of a first session based on the Alcohol, Smoking and Substance Involvement Screening Test (ASSIST)-linked brief intervention [30] delivered using a motivational interviewing style, which can be offered as a stand-alone intervention, with an additional two sessions of problem-solving therapy. Facility-based counsellors implemented the programme. They had all completed high school and were trained for one week on the SBIRT programme. They also participated in weekly supervision conducted by a registered counsellor, which included support for the counsellors, case discussions and ongoing refresher training. Patients who were not seriously ill or injured were approached in EC waiting areas as they waited to see a doctor. In the first year of the programme, 13,136 patients were screened and 37% met criteria for risky substance use on the ASSIST [31] and were offered the programme. Of those meeting criteria, 4005 (83%) received the first session at the acute EC visit and 93 additional sessions were conducted.

### Study design

This study is a mixed methods study, incorporating quantitative screening and patient satisfaction data collected routinely within the SBIRT service, as well as qualitative semi-structured interviews with EC patients who received the programme. The SBIRT programme is described in more detail in van der Westhuizen, Myers et al. (2019), along with process data and perspectives of provincial stakeholder, district stakeholder and hospital staff on the acceptability, appropriateness and adoption of the programme.

### SBIRT service data collection procedures

At the acute visit, patients were approached by a facility-based counsellor who collected basic sociodemographic, hospital presentation and substance use data from consenting patients. Frequency of substance use and associated harms were evaluated using: (i) number of substance use days in the preceding two weeks and (ii) the ASSIST. The ASSIST tool was designed to detect risky substance use, and identify the level of risk (low, moderate, or high risk) for substance-related harms. The tool elicits information regarding the use of tobacco, alcohol, and a full range of illicit drugs over the preceding three months [32]. Substance involvement scores were calculated for each substance and for this programme, scores greater than 6 for alcohol or 1 for drugs were used to identify patients at moderate or high risk of substance-related harms. These cut-off scores were informed by a previous EC study investigating the psychometric properties of the ASSIST [33].

Approximately three months after the acute EC visit, 273 patients were followed up via telephone by a staff member who had not been involved in the SBIRT service. The staff member conducting the telephone calls was mostly available during weekday working hours. Of the 4011 patients who received the programme, 2758 provided telephone numbers, of which 90 numbers were recorded incorrectly. Reasons for not providing contact details were not collected routinely. In some cases, the counsellors indicated that the patient did not own a telephone. The follow-up questionnaire included the number of substance use days in the preceding two weeks (as the primary substance use outcome) and the ASSIST as well as questions on patient satisfaction with services, additional counselling needs and barriers to attending further counselling sessions.

### Study procedures for qualitative interviews

We also conducted semi-structured individual interviews with 18 patients (nine men and nine women between the ages of 20 and 57 years) who had been screened and had received at least the initial session of the SBIRT programme. Eleven of these patients had presented for care of an injury at the acute visit. Patients who had received services from one of two demonstration sites were contacted, given information about the study and invited for an interview at the healthcare facilities. At the time of the interview, the study was explained and informed consent taken. Patients were asked to describe their experience of being approached by the counsellor and receiving the intervention at their acute EC visit, and how the programme could be improved. Furthermore, they were asked to elaborate on any increase or decrease in their substance use as a result of the intervention and the effects of such changes on their daily lives. Their perceptions of barriers and facilitators to attending further sessions were elicited. All interviews were audio-recorded and transcribed.

### Data analysis

Quantitative data were imported into the Statistical Package for the Social Sciences (SPSS) version 25 for analysis. Descriptive statistics were conducted by sex to assess the: (i) proportion of patients presenting with injuries; (ii) the proportion of patients using alcohol or other substances with moderate or high-risk for substance-related harms at the acute visit; (iii) frequency of substance use and (iv) levels of patient satisfaction with the service delivered. Patient characteristics presented in Table 1 were compared by sex using chi-square tests and characteristics were also analysed by age category used chi-square tests. For this study the primary substance was defined as the substance or substances with the highest ASSIST scores for a particular patient. In some cases, patients scored equally high scores for two substances (n = 190) or three substances (n = 40) and these substances were all used as ‘primary’ substances in analyses. For the analysis of substance use outcomes, data were extracted for patients with substance use data from the acute visit and the follow-up call (n = 273). Related sample Wilcoxon signed rank tests were used to compare substance use days and ASSIST scores of participants’ primary substance(s).

For the qualitative component of the study, transcribed data were imported into NVivo version 12 for analysis. We used an inductive approach to the data along with the framework approach [34]. The following steps were performed: (i) familiarisation, (ii) identification of a thematic framework, (iii) indexing, (iv) charting and (v) mapping and interpretation. Two investigators coded a sample of the transcripts. Cohen’s kappa was used to determine the inter-rater reliability with the aim of achieving a kappa of 80% or above. A kappa of 0.94 indicated high inter-rater reliability.

## Results

### Patient characteristics for those meeting criteria for risky substance use

Patients who were using substances at risky levels (n = 4847) were eligible for the brief intervention delivered in the EC. The majority of these patients were male (74%) and almost two thirds of the sample presented with an injury. See Table 1 for the demographic, presenting complaint and substance use characteristics of patients using substances at risky levels. There were statistically significant differences between the male and female groups across all variables, apart from age and unintentional injuries. A greater proportion of male patients used substances and presented with intentional injuries, road traffic crashes and self-harm as compared to female patients. Further, a greater proportion of EC patients aged 35 years and younger suffered any injury (n = 2 249; 70% vs n = 847; 53%), assault injuries (n = 1 647; 51% vs n = 537; 34%) and self-harm injuries (n = 72; 2% vs n = 20; 1%) as compared to those patients older than 35 years. Further, a higher proportion of older EC patients used alcohol only (n = 1 270; 79%) as compared to younger EC patients (n = 2025; 63%), while more younger patients used drugs only (n = 678; 21% vs n = 211; 13%) and alcohol and drugs (n = 532; 16% vs n = 120; 8%) than their older counterparts. These differences were statistically significant. At the acute EC visit, 3707 patients (76%) used alcohol as their primary substance, 794 (16%) used cannabis, 342 (7%) used methamphetamine and 180 (4%) primarily used Mandrax (methaqualone). Of those patients with 2 substances being primary substances (i.e. at equal levels of risk based on the ASSIST scores), the most common combination was alcohol and cannabis (n = 84), followed by Mandrax and methamphetamine (n = 36). For those with three primary substances, the most frequent substances were cannabis, methamphetamine and Mandrax (n = 24).

**Table 1:** Eligible patient characteristics at the acute emergency centre visit

	Total (n = 4847)	Male (n = 3577)	Female (n = 1270)	p-value
Age				0.836
Up to 35 years	3235 (67%)	2381 (67%)	848 (67%)	
Older than 35 years	1601 (33%)	1185 (33%)	416 (33%)	
Injured	3104 (64%)	2434 (68%)	670 (53%)	< 0.001
Intentional (assault)	2188 (45%)	1780 (50%)	408 (32%)	< 0.001
Unintentional (falls, cuts etc.)	581 (12%)	429 (12%)	152 (12%)	0.988
Road traffic crashes	217 (5%)	174 (5%)	43 (3%)	0.029
Self-harm	92 (2%)	30 (1%)	62 (5%)	< 0.001
Alcohol use only	3300 (68%)	2243 (63%)	1057 (83%)	< 0.001
Illicit drug use only	894 (18%)	754 (21%)	140 (11%)	< 0.001
Alcohol and drug use	653 (14%)	580 (16%)	73 (6%)	< 0.001

The three-month follow-up sample (n = 273) was broadly representative of the eligible patient group based on demographics and presenting complaint. However, there were statistically significant differences between the groups regarding substance use measured at the acute EC visit, with 227 (83%) of patients in the follow-up group using alcohol only and not drugs compared to 3071 (67%) in the group who were not followed up. Thus, the follow-up group included far fewer drug users than the original sample.

### Patient substance use outcomes at three-month follow-up

**Fig. 1:**
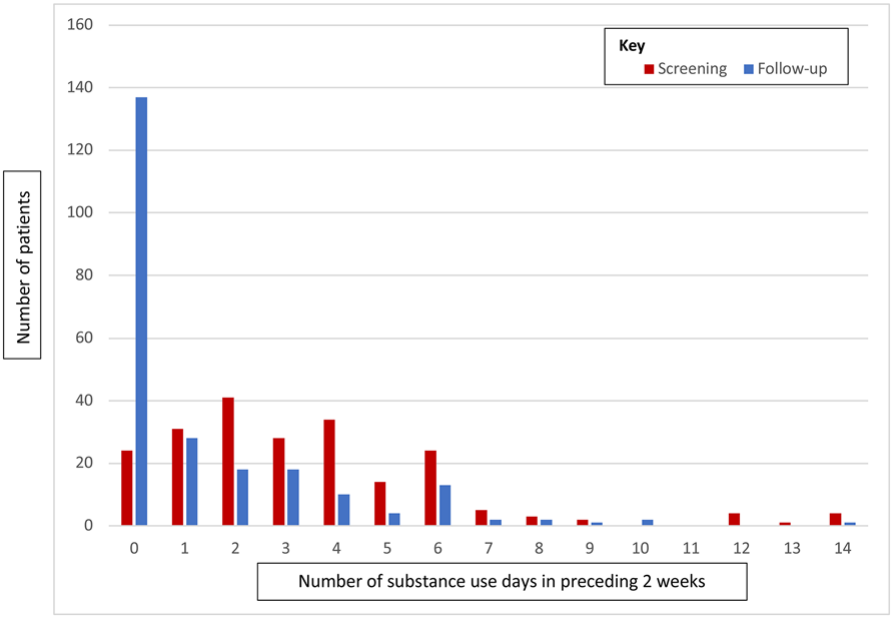
Alcohol use days: screening vs follow-up

For those patients using alcohol as their primary substance (n = 237), the number of alcohol use days in the preceding two weeks decreased significantly (Z = − 7.99; p < 0.001) between the acute EC visit (median = 3; IQR = 4) and the three-month follow-up call (median = 0; IQR = 2) See Fig. 1. Additionally, 138 (59%) of patients reported no alcohol use days at the three-month follow-up, compared to 24 (11%) with no alcohol use days at screening. Similarly, median alcohol scores dropped significantly (Z = − 3.00; p = 0.003) from the acute EC visit interview (median = 13; IQR = 9) to the three-month follow-up interviews (median = 12; IQR = 13).

For patients using drugs as their primary substance (n = 38), the number of drug use days in the previous 2 weeks decreased significantly (Z = − 4.13; p < 0.001) from screening (median = 9.5; IQR = 10) to follow-up (median = 0; IQR = 1.5). At the three-month follow-up, 24 (75%) of patients using drugs reported no drug use days in the preceding two weeks, compared to 3 (9%) at the acute EC visit. ASSIST scores for patients using cannabis (n = 29) and methamphetamine (n = 10) also decreased; although the sample sizes were small. Two patients used Mandrax (methaqualone) as the primary substance, one patient used cocaine and one patient used sedatives.

### Patient satisfaction with services and perceptions of programme acceptability at three-month follow-up

At telephonic follow-up, patients were generally highly satisfied with the services received from the Teachable Moment programme staff, with 97% of patients rating the service as ‘Good’ or ‘Excellent’ at three-month follow-up and 96% of patients being ‘Happy’ or ‘Very happy’ with the amount of help received. Additionally, 97% stated that they would return to the programme if they accessed EC care again. In response to the question, “Do you think that the Teachable Moment Programme should be provided in all ECs in South Africa?”, 98% of the patients completing the follow-up survey responded in the affirmative. The reasons given for this by participants addressed the following broad categories: community benefits (particularly for youth), personal benefits and the service quality. Of the patients followed up who had only attended one session, 73% would have liked to return for further sessions and 53% would have liked to receive telephonic counselling. The most common barrier reported (58%) was a lack of time or money to travel to the hospital, which services a number of communities in a large geographical area. Over a third of patients who reported that they did not want to return for further sessions had decreased or stopped their substance use or felt equipped with sufficient knowledge to manage their use.

Most patients interviewed at the three-month follow-up found the programme acceptable although one patient wasn’t sure “if people would love to listen to the information”, while others highlighted the need for awareness of the risks associated with substance use as well as safe drinking limits for adults.

### Acceptability

Many of the patients interviewed in the qualitative interviews found the screening and intervention at the acute visit “helpful”, reporting that they were “thankful” and that the programme “changed and saved my life at the same time”. Three participants were not able to give an opinion on the programme as they did not remember seeing the counsellor. A few patients mentioned that they appreciated the contact with the counsellor, despite the pain and discomfort they were experiencing at the time; although one patient did mention that she found it difficult to concentrate as her “dress was so red with blood”. This patient later returned to the counsellors and completed all three sessions.

While participants were eager to describe the benefits of the session at the acute EC visit, few took advantage of the additional sessions, with only 93 additional sessions being conducted during the first year of the programme. Among patients completing the three-month follow-up, 265 patients (97%) had received session one only, three (1%) had received two sessions and five (2%) had accessed all three sessions. Some participants felt that they had improved and did not see the need for more sessions:

“I did not want any more because I was so free that time, you see? I did not see any point for me to come back because like okay –from that day I told myself okay … let me change my ways, you see?” *Female patient with medical complaint (participant 5)*

### Acceptability of teachable moment programme components

Certain components of the programme were identified by patients as adding to the positive patient experience. These included the non-judgemental, caring style of the counsellors as well as the screening and psychoeducation offered by the counsellors during the initial session.

#### Counsellor style

Central to the acceptability of the programme was the opportunity to talk with a non-judgemental counsellor in a space where patients felt “free” and accepted, as well as confident that their personal problems would not be shared in their communities. This safe space created by the counsellors allowed participants to feel connected to their counsellor, with one participant referring to his counsellor, as “the mother of the counsel”. Furthermore, the counsellors’ respectful, caring approach was highlighted as a vital aspect of the intervention, which not only encouraged their patients to disclose their problems, but also left patients feeling “uplifted”, “comfortable” and calm, as described by a participant:

“When I was finished here … just chatting here and him explaining a bit, made me feel much better. Look, when I walked out of here, I felt excited about life again.” *Male patient with medical complaint (participant 10)*

Additionally, participants recommended that others also access the programme if they “want to be helped” and that if they are “a good listener [open to hearing what the counsellor says], this programme will help you a lot and give you a second chance in life”. For the programme to be successful, participants stressed the importance of taking responsibility for life choices and making a personal decision to decrease substance use:

“If you go there to the counsellor, go with your lovely heart and then tell yourself: I can change.” *Female participant with an assault injury (participant 18)*

#### Screening and psychoeducation

In addition to participants appreciating the safe space to talk, certain aspects of the programme were particularly beneficial, with participants describing ‘teachable moment’ experiences in the counselling room. The substance use screening, personalised feedback about their risk of harm, and psychoeducation facilitated these experiences:

“I needed someone or something that will ask me these questions so that I can also balance myself. These questions, I have never asked them–I never asked myself these questions, but someone did ask me these questions and then I started to realise, I need help, I really need help, you see.” *Male patient with assault injury (participant 1)*

While several participants mentioned being “caught off guard” by being approached and screened by a Teachable Moment counsellor, this component was generally very well-received; although one participant who reported that the screening was acceptable, also mentioned wondering why “someone that doesn’t know me is asking me such things”.

### Perceived benefits of the teachable moment programme

The patients reported benefiting from the programme in various ways. First, most participants reported decreasing substance use, with more than half of the participants stating that they had stopped using alcohol completely. Others spoke about how they had cut down on drinking days and the quantity of alcohol consumed. These reports were similar to the changes reported during the follow-up telephone calls. Of the patients participating in the qualitative interviews, two participants mentioned drug use, one of whom had stopped using drugs after attending both the Teachable Moment programme as well as another community outpatient substance programme. Another patient reported decreasing his cannabis use to once a week and then only having “two or three pulls”.

Participants described implementing a number of strategies to decrease or avoid using substances all together. The strategies addressed triggers identified in counselling sessions, with stress, social spaces and ‘drinking friends’ being the most commonly mentioned triggers. Participants tackled these triggers by either planning alternative activities, spending time with family, avoiding ‘drinking friends’ or consuming soft drinks while others were using alcohol. It was especially difficult for some to avoid substance use on weekends. One participant recounted her weekend strategy:

“Today is Friday. I must go to drink then I said, ‘Uh-uh! It is all the devil’s work …’ On Fridays I am working. I go to work about three o’ clock in the day. I also thank God that I am busy on the weekend so that I am not going to think about alcohol.” *Female patient with assault injury (participant 11)*

Second, along with the decrease in substance use, participants reported improvements in health. They perceived these improvements as significant benefits. Apart from describing overall benefits to wellbeing, participants also described benefits in terms of injuries to themselves and others. One participant described how they no longer feel the urge to “beat my friends when I am drinking”. Participants spoke of their past drinking behaviour and the associated health consequences to illustrate the positive health benefits of decreasing their alcohol use, saying that in the past their alcohol use was “destroying my body”.

Third, participants highlighted the benefits realised in their interpersonal relationships. Participants reflected how their previous substance use behaviour had impacted their interpersonal relationships, causing family conflict and, in some cases, negatively affecting their parenting. After participating in the Teachable Moment programme, participants spoke about prioritising time with their children, partners and other family members, using the time that they would usually have spent drinking with friends and being a “party animal”. While participants acknowledged that they had lost friendships or saw their friends much less, this wasn’t always seen as a negative aspect. Participants reported enjoying time with the family, which they had not experienced before, as one participant described:

“I have seen now if I stay with my family we just watch the TV or I am cooking. Mommy is cooking there … just chatting with my parents. I did not feel that, did not know that it is something nice. But now, I am staying next to my family and I just want to continue doing that.” *Female patient with unintentional injury (participant 9)*

Fourth, participants reported that an added benefit of decreasing their substance use was having “money in the bank” to spend on their children and household necessities. Consequently, some participants reported that they no longer needed to borrow money, avoiding stress and high interest rates. Furthermore, participants mentioned beginning to think about life goals to increase their earnings and provide for their futures, such as finding employment or educational opportunities. Participants spoke about moving their lives forward, which some felt was not possible before when they were drinking.

### Recommendations for improving the SBIRT programme

Study participants made recommendations regarding: (i) changes to the session format, (ii) extending the reach of the programme to include a broader age group and a variety of settings, and (iii) marketing of the programme.

Participants suggested that the programme should be extended to include younger people, highlighting the need for substance use information and intervention for adolescents because “the kids are spending time drinking, fighting”. They also spoke of other problems associated with adolescent alcohol and drug use in their communities, such as adolescents being murdered, committing crimes and ending up in prison, contracting HIV and young girls becoming pregnant. Participants mentioned the need for prevention and early intervention for risky substance use as adolescents do not realise that “you feel nice at that moment, but as time goes by, that becomes a major problem in your life”.

While many participants in the follow-up and qualitative interviews urged the team to “keep on doing the good work” in the ECs and expand the programme to include “all clinics and hospitals”, they also indicated that the counsellors should not just “wait for people in hospital” but should include additional locations in the community. Locations suggested included schools, churches, community centres, malls “and even in playgrounds”. To achieve increased reach, a number of marketing strategies were recommended. Some strategies involved little contact with the public, such as placing “posters on the street, in the [taxi] ranks, bus stops”, and handing out pamphlets with substance use information and details on how to access help. More active forms of marketing the programme were also suggested, such as speaking to people in public places facilitated by just putting “your tables there on the pavement”. Further strategies included attracting people to an information session by using vehicles with loudspeakers and music, and providing food and games.

## Discussion

This is one of the few studies exploring patient experiences of a task shifted SBIRT programme within EC settings in an LMIC and provides some key findings. First, substance use screening and intervention by facility-based counsellors appears acceptable to patients in an LMIC EC setting. Second, screening and one motivational-interviewing based session, which includes personalised feedback on substance use scores, was reported to be beneficial, with patients perceiving improved substance use outcomes and interpersonal relationships as well as economic benefits. Third, counsellors’ caring, non-judgemental style and supportive approach is central to patients’ good opinions of SBIRT. Fourth, the main recommendation offered by participants was to extend the programme to include adolescents and community settings outside healthcare facilities, as well as market the programme and disseminate health information.

Patients found the SBIRT programme acceptable and were satisfied with the service they received while waiting for medical care. This is supported by data on the proportion of patients at risk of substance-related harms completing a session at the acute EC visit (83%) as reported in a previous paper [13], and by the findings of the original randomised controlled trial of the intervention in South African ECs [24]. These findings are similar to reports of SBIRT programmes delivered by professional healthcare workers, such as non-specialist doctors and nurses [23, 35, 36], and adds to the body of literature endorsing the acceptability of community healthcare worker-delivered mental healthcare interventions [37].

In this study, a single session with the Teachable Moment counsellor was well received, with several participants reporting that they did not see the need to return for further sessions, mainly due to various barriers, such as time and resources, and many felt that they had already received the benefits of the programme. These reports of patient experiences and outcomes were in keeping with the substance use reductions seen in the followup screening data. The most valuable aspects from the patients’ perspectives were the substance use screening and information, and personalised feedback on substance use risk level. These aspects are common in other SBIRT programmes, and are often delivered utilising a motivational interviewing style, as in the Teachable Moment programme [38]. In evaluation studies including SBIRT recipients in various healthcare settings, the screening and feedback was seen as helpful by the majority of patients receiving the programme [24, 39, 40]. Additionally, the barriers to attending further sessions reported in this study are commonly cited factors influencing access to mental health and substance use care in LMICs [41, 42]. In the future, these barriers could be overcome by offering access to digital or telephonic interventions, which have shown promise in intervening for risky substance use [43, 44]. However, although smartphone and internet access is increasing in LMIC settings [45], access may be limited in the substance-using population presenting for emergency care.

In practice, multiple sessions are infrequently accessed, particularly in emergency or trauma care settings [46]. In SBIRT programmes operating in healthcare settings across 4 states in the USA, clients received on average 1.2 sessions of a brief intervention [47] and those accessing brief treatment received an average of 2.6 sessions. In these programmes, brief interventions comprise between one and five sessions, lasting from 5 min up to an hour, while brief treatment programmes offer between five and twelve longer and more intensive sessions. In a comparison between brief intervention and brief treatment services, there was minimal difference in outcomes for people who use alcohol at 6-month followup, suggesting that a single session may be sufficient for reducing risks in those using alcohol at unhealthy levels [47]. Furthermore, brief intervention produced improved substance use outcomes at far less cost than brief treatment services, although it is not known if these benefits were sustained beyond 6 months.

In this study the caring, supportive style of the counsellors was central to patients’ positive perception of the SBIRT service. While few other studies have included SBIRT recipients in evaluation research and interrogated the style of the counsellor, some studies report SBIRT recipients appreciating being able to speak with a non-judgemental SBIRT provider [25, 39]. Although we didn’t examine the association between recipients’ perception of the counselling relationship and outcome, there is evidence from other studies that counsellor empathy and collaboration is associated with better client outcomes [48, 49]. In some substance use intervention studies, empathy was the only counsellor competency domain that predicted better outcomes [50].

Patients recommended extending the reach of the SBIRT programme, which included suggestions for additional community intervention sites, including adolescents, marketing the programme and spreading information on problem substance use. Similar suggestions have been made by participants in studies exploring SBIRT acceptability before and during implementation, highlighting the need for engaging community leaders and the general public to discuss substance use problems and the need for interventions [51]. During SBIRT implementation in the USA funded by the Substance Abuse and Mental Health Services Administration Center for Substance Abuse Treatment across multiple states, ongoing evaluation and feedback was employed to improve services and implementation strategies from one cohort of grantees to the next. In later cohorts, efforts were expanded from healthcare settings only to include additional community settings which allowed grantees to provide services to a much larger population with a more varied substance use risk profile [52].

Future research in South African settings should ideally adopt an implementation science approach and evaluate strategies to extend substance use brief interventions into community settings. Such strategies could include leveraging existing community programmes and services, such as youth and sports programmes, and libraries where young people often gather to access the internet for recreation, study or job-seeking. Additional platforms such as telephone or web-based interventions should also be explored, particularly where free access could be facilitated, such as through toll-free helpline numbers.

### Limitations

While this study provides some valuable findings regarding a facility-based counsellor-delivered SBIRT programme in an LMIC setting, there are limitations to the research. First, it was not possible to include a control group of healthcare facilities due to the rapid implementation of the SBIRT programme into usual care and due to the limited available funding. Patient substance use outcomes were investigated using a pre-post study design and while these outcomes showed a statistically significant improvement for those using alcohol in keeping with other SBIRT studies, these results should be interpreted with caution. Furthermore, at the acute EC visit only 252 patients (5%) screened high risk for alcohol use and 19 of these patients (7% of the followup sample) were followed up at three months. These individuals are more likely to require specialised services and it is not possible to draw conclusions regarding intervention for this group based on this study. Second, the limited number of patients accessed for the follow-up telephonic interview also influences the strength of the evidence reported. In other studies conducted by our team and colleagues which recruited people who use substances, intensive procedures were used to improve retention in the study, which include travelling to communities to locate participants in their homes or public places [53, 54]. These procedures were not feasible for this evaluation of a routinely implemented service. Third, although the study found improved substance use outcomes in the follow-up sample and additional patients reported SBIRT benefits during qualitative interviews, these findings may not be generalisable to the full population accessing the SBIRT programme as those who benefited could be more likely to participate in follow-up research.

## Conclusion

This task-shared SBIRT service was found to be acceptable to patients, who reported multiple benefits of a single SBIRT contact session delivered during an acute EC visit. These findings add to the SBIRT literature by highlighting the role of non-professional healthcare workers in delivering a low-intensity SBIRT service feasible to implement in low-resourced settings. Additionally, findings suggest that implementers should ensure that counsellors employ a non-judgemental approach, and focus on screening, feedback and psychoeducation as key components of a SBIRT service.

## Data Availability

The data that support these findings are owned by the Western Cape Department of Health and applicants may apply online to the National Health Research Database (https://nhrd.hst.org.za/).

## Abbreviations

ASSIST: Alcohol, Smoking and Substance Involvement Screening Test
EC: Emergency centre
LMIC: Low-and middle-income country
SBIRT: Screening, brief intervention and referral to treatment
SPSS: Statistical Package for the Social Sciences
